# Broadly neutralizing monoclonal antibodies for HIV prevention

**DOI:** 10.1002/jia2.25829

**Published:** 2021-11-21

**Authors:** Maurine D. Miner, Lawrence Corey, David Montefiori

**Affiliations:** ^1^ Vaccine and Infectious Disease Division Fred Hutchinson Cancer Research Center Seattle Washington USA; ^2^ Department of Surgery and Duke Human Vaccine Institute Duke University Medical Center Durham North Carolina USA

**Keywords:** AMP, bnAb, HIV prevention, monoclonal antibody, TZM‐bl assay, VRC01

## Abstract

**Introduction:**

The last 12 years have seen remarkable progress in the isolation and characterization of at least five different epitope classes of HIV‐specific broadly neutralizing antibodies (bnAbs). Detailed analyses of these bnAb lineages, maturation pathways and epitopes have created new opportunities for vaccine development. In addition, interest exists in passive administration of monoclonal antibodies as a viable option for HIV prevention.

**Discussion:**

Recently, two antibody‐mediated prevention (AMP) trials of a passively administered monoclonal antibody targeting the HIV envelope CD4 binding site, called VRC01, provided proof‐of‐concept that monoclonal antibody infusion could offer protection against HIV acquisition. While the trials failed to show overall protection against HIV acquisition, sub‐analyses revealed that VRC01 infusion provided a 75% prevention efficacy against HIV strains that were susceptible to the antibody. The study also demonstrated that in vitro neutralizing activity, measured by the TZM‐bl/pseudovirus assay, was able to predict HIV prevention efficacy in humans. In addition, the AMP trials defined a threshold protective concentration, or neutralization titer, for the VRC01 class of bnAbs, explaining the observed low overall efficacy and serving as a benchmark for the clinical testing of new bnAbs, bnAb cocktails and neutralizing antibody‐inducing vaccines. Newer bnAbs that exhibit greater potency and breadth of neutralization in vitro than VRC01 are available for clinical testing. Combinations of best‐in‐class bnAbs with complementary magnitude, breadth and extent of complete neutralization are predicted to far exceed the prevention efficacy of VRC01. Some engineered bi‐ and trispecific mAbs exhibit similar desirable neutralizing activity and afford advantages for manufacturing and delivery. Modifications that prolong the serum half‐life and improve genital tissue persistence offer additional advantages.

**Conclusions:**

Iterative phase 1 trials are acquiring safety and pharmacokinetic data on dual and triple bnAbs and bi‐ and trispecific antibodies in preparation for future AMP studies that seek to translate findings from the VRC01 efficacy trials and achieve acceptable levels of overall prevention efficacy.

## 1 INTRODUCTION

Despite tremendous effort for the past 30 years to develop an effective HIV vaccine, the field remains challenged. The HIV envelope protein (Env), found on the surface of virions and the major target of neutralizing antibodies, is highly genetically diverse, covered by a glycan shield, and expressed at a relatively low density [1]. Over the last decade, there has been remarkable progress in the isolation and characterization of several different classes of HIV‐specific broadly neutralizing antibodies (bnAbs), defined by their ability to neutralize multiple genetically distinct strains. There are essentially five regions of Env that neutralizing antibodies bind: CD4 binding site (CD4bs), variable loop 2 (V2)‐apex, V3‐glycan, glycoprotein (gp)41/gp120 interface, and membrane proximal external region (MPER) [2, 3, 4, 5, 6, 7]. Antibodies targeting all of these sites have been identified, each analyzed for its breadth (number of viral strains it can neutralize) and potency (concentration required for neutralization).

The breadth/potency of these bnAbs fostered the idea of passive administration of monoclonal antibodies (mAb) as an option for HIV prevention, a technique used to prevent respiratory syncytial virus in high‐risk infants and, most recently, COVID‐19 [8, 9]. Advances in next‐generation sequencing and B‐cell cloning have led to numerous potential bnAbs for efficacy testing. The recently published antibody‐mediated prevention (AMP) trials jointly conducted by the HIV Vaccine Trials Network (HVTN) and HIV Prevention Trials Network (HPTN) demonstrated the feasibility of this approach [10]. This commentary focuses on recent results from the first efficacy trials testing bnAbs for HIV prevention and provides a roadmap to move the field forward.

## 2 DISCUSSION

### 2.1 VRC01 proof‐of‐concept AMP trials

VRC01, an antibody isolated and characterized from an individual with immunologically controlled HIV, targets the Env CD4bs [11]. VRC01 can neutralize a large percentage of HIV reference strains in vitro using a pseudovirus neutralization assay (see subsequent section) [12, 13] and protect against infection in nonhuman primates (NHPs) [14, 15, 16, 17, 18]. Several concentrations of VRC01, up to 40 mg/kg, delivered intravenously (IV) were safe and non‐immunogenic in HIV‐uninfected adults and decreased plasma viral load people living with HIV [19, 20, 21]. Recent analyses of participants from a phase 1 trial (who received either 10 or 30 mg/kg VRC01) identified VRC01 in genital tissue, and tissue explants were protected from ex vivo HIV challenge [22]. As such, the proof‐of‐concept phase 2b AMP studies were designed to test whether VRC01 could prevent HIV infection. AMP participants received either 10 mg/kg VRC01, 30 mg/kg VRC01 or placebo IV every 8 weeks for a total of 10 infusions. These studies were carried out in populations at high risk of acquiring HIV: 2699 men and transgender women who have sex with men in the Americas and Europe (HVTN 704/HPTN 085) and 1924 heterosexual women in sub‐Saharan Africa (HVTN 703/HPTN 081) [23, 24]. The studies also aimed to establish whether a pseudovirus neutralization assay using TZM‐bl cells was a reliable biomarker of HIV prevention and to identify a threshold serum neutralization titer required for protection.

Analyses of the AMP trials indicated that while VRC01 did not provide overall efficacy against HIV acquisition, pre‐specified analyses indicated a 75% prevention efficacy (PE) against strains that were susceptible to VRC01 [10]. This PE was irrespective of gender, route of transmission, or viral subtype, indicating the innate susceptibility/resistance of the circulating strains to the antibody was the primary determinant of efficacy. While viruses with this level of susceptibility accounted for only 30% of circulating strains at the trial sites [10], the study indicated that bnAbs, when sufficiently potent against infecting viruses, can prevent HIV. As such, subsequent antibodies need to be broader and more potent than VRC01. Another hallmark finding from the AMP trials was that the in vitro TZM‐bl neutralization assay was predictive of in vivo HIV sensitivity to antibody. This finding sets the stage for testing new mAbs/mAb cocktails iteratively to down select clinical trial contenders and allow accelerated development of higher potency and more easily manufactured antibodies.

### 2.2 Evolution of HIV neutralization assays and reference strains

Neutralization assays with Env‐pseudotyped viruses and stable engineered target cells, together with well‐characterized reference strains, were transformative and immensely valuable in the discovery and in vitro characterization of bnAbs leading up to the VRC01 AMP trials. A plethora of assays and virus strains used early in the pandemic (1984–2003) produced conflicting results. Attempts to understand these contradictory data pointed to differences in the cells used for virus production and infection target, type of serologic reagent employed (e.g. serum or mAb), and, perhaps most importantly, viral strain used [25, 26]. The gold standard assay for many years utilized viruses grown and assayed in peripheral blood mononuclear cells with the assumption that this format best mimicked natural infection; however, these assays were highly variable, low throughput, labour intensive and resisted intensive efforts to standardize. The pseudovirus technology that emerged in 2003 [27, 28] afforded superior sensitivity, precision and high throughput capability. Moreover, clonal Envs with distinct sequences enabled detailed functional studies of neutralization epitopes and their escape pathways. A consensus in 2004 emphasized the value of the pseudovirus technology and recommended that well‐characterized panels of Env‐pseudotyped viruses be developed as standard reference reagents [29]. Subsequently, the Env‐pseudotyped virus assay in TZM‐bl cells (TZM‐bl assay) [30] used in the AMP trials was optimized, qualified, validated [31] and transferred to multiple laboratories around the world [32]. In addition, a formal proficiency testing program was implemented in 2009 to evaluate equivalency of TZM‐bl assay performance across multiple laboratories [33]. These efforts improved the accuracy and comparability of results to enable aggregate bnAb datasets to be evaluated with confidence.

Early HIV neutralization studies also suffered from a lack of understanding of the virus. Although a deep appreciation existed for Env sequence diversity, little knowledge was available on the molecular structure and conformational plasticity of native Env trimers. Studies revealed that native‐like Env trimers spontaneously transition through open, closed and intermediate conformations, with the open conformation exposing immunodominant epitopes to a greater extent than when the trimer is in an intermediate or closed conformation [34, 35, 36]. These conformational states have profound effects on the neutralization phenotype of the virus. An open conformation explains why early studies with T‐cell line adapted strains grossly overestimated vaccine‐elicited responses [37, 38, 39]. Most circulating strains exhibit a more closed trimer conformation that is not susceptible to the bulk of easily induced Env‐specific antibodies. To help distinguish these properties, a tiered categorization of HIV‐1 isolates was developed based on their neutralization phenotype when assayed with polyclonal HIV‐1 sera; from most promiscuously neutralized (open trimer; tier 1; rarely in circulation) to harder‐to‐neutralize (intermediate/closed trimer; tiers 2 and 3; majority of circulating strains) [35, 40].

Hundreds of reference Envs representing all major genetic subtypes and circulating recombinant forms are available and in wide use as pseudotyped viruses for neutralization assays [41, 42, 43, 44, 45, 46]. As new bnAbs were discovered and assayed against large panels of reference strains, the magnitude and breadth of bnAb activity was, and continues to be, compared and used to prioritize candidates for clinical development. Testing combinations of bnAbs for possible synergy is also of interest. One large study examined bnAbs targeting four different epitopes (CD4bs, V2‐apex, V3‐glycan and MPER) in all possible dual, triple and quadruple combinations against a panel of 125 Env‐pseudotyped viruses and found the combined effects were mostly additive, being explained by the complementary neutralization profiles of the individual bnAbs [47]. Based on these additive effects, and using raw data from the study, a Bliss–Hill model was developed that enabled the neutralization magnitude and breadth of various bnAb combinations to be predicted using single bnAb data [48]. Use of the Bliss–Hill model accelerated the identification of optimal bnAb combinations to explore for HIV prevention and treatment without the need for time‐consuming and labour‐intensive experimental testing of all possible combinations against large panels of viruses [48, 49, 50]. Viruses from infected placebo recipients in the AMP trials, representing more contemporary genetic and antigenic diversity, are additional useful reference reagents for single and combination bnAb evaluation.

### 2.3 Estimation of protective neutralization titers for future bnAb trials

When the first‐in‐human VRC01 trials were designed, VRC01 neutralization, measured by the in vitro 50% inhibitory concentration (IC_50_), surpassed 90% of viral reference strains at an IC_50_ <50 mcg/ml and 72% of strains at an IC_50_ <1 mcg/ml (or 86% at an IC_80_ <50 mcg/ml and 42% <1 mcg/ml) [12, 13]. When administered at 10 mg/kg, for example, the in vivo VRC01 trough levels were predicted to be 4 mcg/ml, which corresponds to a neutralization breadth based on IC_50_/IC_80_ values of 94%/93% against subtype B strains and 80%/73% against subtype C isolates [51]. Results from the AMP trials permitted an estimation of protective serum neutralization titers in vivo (50% and 80% inhibitory dilutions, or ID_50_ and ID_80_). ID_50_/ID_80_ titers were calculated by dividing the median mid‐infusion VRC01 concentration across the 10 infusions by the IC_50_ or IC_80_ of VRC01 against the participant's acquired virus. Achieving 50%, 75% and 90% PE was predicted to require ID_50_ titers of 1:116, 1:252 and 1:565, respectively, with corresponding ID_80_ titers of 1:32, 1:82 and 1:194 (Gilbert et al. submitted). With this information in hand, it now is possible to design future mAb trials to achieve such neutralizing titers and, thus, clinical efficacy.

### 2.4 Comparison of NHP and human data

The rationale for the VRC01 AMP trials was based in part on studies from several laboratories showing that mAbs against multiple Env epitopes could protect against high‐dose intrarectal and intravaginal SHIV challenge in NHPs. Early NHP studies showed that neutralizing titers of 50–100 were required for 50% protection [18] and that V2‐apex and V3‐glycan‐specific antibodies protected at remarkably low titers, consistent with their high in vitro neutralizing potencies [52, 53]. The AMP study serum neutralization titers now allow validation of NHP challenge models for preclinical predictive efficacy.

To date, there are many similarities between the data derived from NHP high‐dose mucosal challenge models and the AMP trials. A meta‐analysis of 17 studies involving 274 NHPs and all classes of bnAbs showed that neutralizing activity in serum at the time of challenge was the primary determinant of protection irrespective of challenge virus, route of infection or mAb epitope specificity [54, 55]. ID_50_ titers of 1:91, 1:219 and 1:685 were required for 50%, 75% and 95% protection, respectively, which is remarkably similar to titers predicted from the AMP trials mentioned above. These data suggest that neutralization titer in serum has the potential to be a surrogate marker for predicting PE similar to viral load RNA in plasma as a predictor of antiretroviral efficacy. This observation also has important implications for defining the serum titers required for vaccines intended to elicit protective antibodies.

A major priority now is to develop one or more mAb cocktails or multi‐specific mAbs that provide high‐level (>80%) protection against HIV acquisition in multiple geographic regions and risk groups. This goal is likely achievable, on the basis of the breadth and potency of current mAbs under study and their ability to prevent SHIV infection in NHPs [14, 16, 18, 52, 53, 56, 57, 58, 59, 60, 61, 62, 63, 64]. For example, a recent study showed a mAb cocktail was required for complete protection, as single antibodies failed to protect due to differential resistance profiles of the challenge viruses [62]. These data support the notion that mAb cocktails can enhance PE against diverse HIV strains.

One caveat to using the meta‐analysis data is that each NHP experiment used a homogeneous SHIV stock as the challenge virus [54], which is distinct from viral swarms that humans are typically exposed to. In most of these studies, each NHP was administered one bnAb followed by challenge with a single viral strain. Going forward, NHP studies should use strain mixtures to more accurately reflect what occurs during human transmission and provide a better comparative strategy for designing combinations of bnAb cocktails for HIV prevention.

### 2.5 bnAbs with greater potency and breadth

As the VRC01 concentration predicted to protect against HIV was insufficient for the majority of circulating strains observed in the AMP trial, future mAbs/mAb cocktails will need wider in vivo coverage and higher potency [63, 64]. As with first‐generation antiretrovirals, resistance to drug abrogated single drug efficacy and stimulated the use of drug cocktails. The same will most likely be required for mAb prevention strategies.

As the AMP study was ongoing, a series of phase 1 trials were designed and conducted to determine the best antibody or antibody cocktail to move forward clinically. Table 1 lists the current or planned clinical trials for single, double or multiple mAb cocktails, which display a broad range of neutralization potency and breadth [19, 65, 66, 67, 68, 69, 70]. Great strides have been made in improving upon both breadth and potency [71, 72, 73] as well as manufacturability, stability, epitope affinity and serum half‐life through antibody screening and targeted engineering [2, 74, 75, 76]. For example, antibody recycling and turnover is regulated through Fc binding the neonatal Fc receptor, which then limits lysosomal degradation, and modifications to the Fc region can prolong plasma half‐life [77]. A leucine and serine double mutation in the Fc region of VRC01, VRC01‐LS, increases serum half‐life and genital tissue persistence [15, 78]. The LS mutation has been subsequently engineered into many anti‐HIV bnAbs for clinical trial testing (Table 1).

**Table 1:** Clinical status of the mAb pipeline for HIV prevention trials

Antibody	Target	Clinical trial completed	Ongoing/in development
VRC01	CD4bs	NCT02568215, NCT02716675, NCT02797171, NCT03831945, NCT02579083, NCT02165267, NCT01993706, NCT01950325, NCT02471326, NCT02664415, NCT02411539, NCT03208231, NCT02463227	NCT02256631, NCT02140255, NCT04860323, NCT04801758, NCT02591420
VRC01‐LS	CD4bs	NCT02797171, NCT02599896, NCT02840474	NCT03707977, NCT02256631
VRC07‐523‐LS	CD4bs	NCT02840474, NCT03205917, NCT03015181, NCT03387150, NCT03565315 (term), NCT03735849,	NCT03928821, NCT04212091, NCT02256631, NCT04357821, NCT04340596, NCT03739996, NCT03721510, NCT03803605
3BNC117 and 3BNC117‐LS	CD4bs	NCT02825797, NCT02824536, NCT03571204, NCT02018510, NCT02850016, NCT03254277, NCT02588586, NCT02446847	NCT04811040, NCT04319367, NCT04720742 (susp), NCT03837756, NCT04173819, NCT03588715, NCT03554408, NCT03526848, NCT04250636, NCT03041012, NCT04819347, NCT04560569
CAP256V2LS	V2‐apex		NCT04408963
PGDM1400 and PGDM1400‐LS	V2‐apex	NCT03205917	NCT03928821, NCT03721510
PGT121 and PGT121‐LS and PGT121.414.LS	V3‐glycan	NCT03205917, NCT02960581	(NCT03928821, NCT03721510, NCT04212091
10‐1074 and 10‐1074‐LS	V3‐glycan	NCT02825797, NCT02511990, NCT02824536, NCT03831945, NCT03571204	NCT03928821, NCT03707977, NCT04340596, NCT04811040, NCT04357821, NCT04319367, NCT04720742 (susp), NCT03837756, NCT03619278, NCT04173819, NCT03588715, NCT03554408, NCT03526848, NCT04250636
SAR441236 (Sanofi trispecific)	CD4bs, V2‐apex, MPER		NCT03705169)
iMAb/10E8v4	MPER		NCT03875209
10E8‐LS	MPER	NCT03565315 (term)	

Note: Trials registered at clinicaltrials.gov. Includes trials in healthy adults, people living with HIV, and HIV‐exposed infants.

Studies considered completed based on clinicaltrials.gov.

Abbreviations: Susp, suspended; Term, terminated.

### 2.6 Optimizing mAb combinations and delivery

There are several considerations for mAb combination strategies. The potency and breadth of mAb combinations are greater than with single antibodies, double or triple coverage targeting multiple epitopes can impede viral escape, and combinations may reduce levels of incomplete neutralization [47, 48, 49, 50]. When tested in vitro against multiclade panels of HIV‐1 pseudoviruses, PGT121 and PGDM1400 displayed limited breadth, but also had complementary coverage and outstanding potency, with among the lowest median IC_50_ and IC_80_ titers among all bnAbs identified to date [63].

In addition to using cocktails, the delivery route of antibodies can be altered. IV infusion allows high mAb bioavailability and has no volume limit, but requires clinic visits and is typically expensive. Subcutaneous (SC) injection, on the other hand, could be done virtually anywhere for lower cost, but results in reduced plasma bioavailability compared to IV and is limited by volume (reviewed in [79]). Co‐administration with hyaluronidase, however, can increase product uptake with SC administration [79]. Of course, even the most potent bnAbs will not effectively lessen the HIV pandemic unless they are available in areas of most need, typically underserved low‐ to middle‐income countries, with global equitable access [80]. Manufacturing cost, cold‐chain requirement, scalability and portable nature of mAb delivery will need to be optimized for implementation even in low‐infrastructure regions (reviewed in [81]).

Bi‐ and trispecific antibodies have been constructed to broaden neutralization coverage into a single manufacturable antibody. The bispecific antibody 10E8‐iMab that targets both Env and host cell CD4 has shown remarkable promise with high potency even at lower concentrations than best‐in‐class single and combination bnAbs across multiple HIV subtypes [50]. The 10E8 antibody has broad coverage, albeit somewhat lower potency, but when combined with the anti‐CD4 mAb ibalizumab, has powerful in vitro activity [82]. A trispecific antibody showing potent activity in NHP [83, 84] has also entered clinical trials (Table 1). One of the major unanswered questions of these artificially conceived therapeutics is their uptake in patients and their durability to maintain effectiveness. Monitoring for antidrug antibodies (ADA) will be an important first step in their early evaluation, as they are more “atypical” than human‐derived antiviral mAbs, such as VRC01, which has not stimulated ADA in trial participants to date.

## 3 CONCLUSIONS

Results from the VRC01 AMP trials and iterative bnAb evaluation over the past decade place us in an opportune time for the future of mAb prevention studies. Our advances in bnAb screening and engineering have drastically increased the potency and breadth of mAbs, identified additional epitopes for neutralization and opened the door for multi‐specificity and mAb cocktails. Moreover, antibody engineering efforts have generated antibody variants with increased half‐lives, potentially enabling administration schedules every 3–6 months and by the SC route, which would be a promising alternative to an antiretroviral‐based prophylaxis regimen, especially in regions with low access to IV infusion clinics. In addition, the AMP trials provided critical insights into the serum neutralizing antibody titer required for protection, which will inform the development of next‐generation HIV vaccines. It will be of utmost importance for scientists in academia, governmental bodies, commercial partnerships and community stakeholders to collaborate as we build off the first bnAb HIV efficacy trials.

## COMPETING INTERESTS

The authors declare no competing interests.

## AUTHORS' CONTRIBUTIONS

LC, DM and MDM developed the outline. MDM wrote the first draft. All authors contributed to writing and editing and approve the final version.

## FUNDING

National Institute of Allergy and Infectious Diseases grant UM1 AI068614.

## Data Availability

All data mentioned are provided in this commentary.
